# Electroacupuncture Prevents Cognitive Impairments by Regulating the Early Changes after Brain Irradiation in Rats

**DOI:** 10.1371/journal.pone.0122087

**Published:** 2015-04-01

**Authors:** Xing-Wen Fan, Fu Chen, Yan Chen, Guan-Hao Chen, Huan-Huan Liu, Shi-Kuo Guan, Yun Deng, Yong Liu, Sheng-Jian Zhang, Wei-Jun Peng, Guo-Liang Jiang, Kai-Liang Wu

**Affiliations:** 1 Department of Radiation Oncology, Fudan University Shanghai Cancer Center, Shanghai, China, 200032; 2 Department of Integrative Medicine and Neurobiology, State Key Laboratory of Medical Neurobiology, Shanghai Medical College, Fudan University, Shanghai, China, 200032; 3 Department of Radiology, Ruijin Hospital, Shanghai Jiaotong University, Shanghai, China, 200032; 4 Department of Radiology, Fudan University Shanghai Cancer Center, Shanghai, China, 200032; 5 Department of Oncology, Shanghai Medical College, Fudan University, Shanghai, China, 200032; University of Queensland, AUSTRALIA

## Abstract

Cognitive impairments severely affect the quality of life of patients who undergo brain irradiation, and there are no effective preventive strategies. In this study, we examined the therapeutic potential of electroacupuncture (EA) administered immediately after brain irradiation in rats. We detected changes in cognitive function, neurogenesis, and synaptic density at different time points after irradiation, but found that EA could protect the blood-brain barrier (BBB), inhibit neuroinflammatory cytokine expression, upregulate angiogenic cytokine expression, and modulate the levels of neurotransmitter receptors and neuropeptides in the early phase. Moreover, EA protected spatial memory and recognition in the delayed phase. At the cellular/molecular level, the preventative effect of EA on cognitive dysfunction was not dependent on hippocampal neurogenesis; rather, it was related to synaptophysin expression. Our results suggest that EA applied immediately after brain irradiation can prevent cognitive impairments by protecting against the early changes induced by irradiation and may be a novel approach for preventing or ameliorating cognitive impairments in patients with brain tumors who require radiotherapy.

## Introduction

Brain irradiation (BI) is a first-line treatment for both primary and metastatic intracranial malignancies. Irradiation-induced cognitive impairments, including dementia, occur in 50–90% of patients with brain tumors who survive >6 months postirradiation [1]. Moreover, the incidence and severity of these impairments increase over time. A benefit of improved radiation therapy techniques and systemic therapies is that patients with brain tumors are surviving longer; however, this means that the patient population with irradiation-induced cognitive impairments is growing rapidly. Quality of life is dramatically affected by cognitive dysfunction and is an important measure of the outcome of brain tumor therapy. Although some-short term interventions are effective [2], there is a lack of useful preventive strategies for irradiation-induced cognitive impairments. Thus, research aimed at preventing or ameliorating irradiation-induced cognitive impairments is important.

Valuable insights have come from preclinical studies regarding potential pathogenic mechanisms involved in radiation-induced cognitive impairment. Radiation to the brain induces a profound oxidative stress and inflammatory response [3,4]. Loss of hippocampal neurogenesis [5], persistent changes in neuronal structure and synaptic plasticity [6], white matter impairment [7], blood-brain barrier (BBB) damage [8], and decreased capillary density [9] have been associated with radiation-induced cognitive impairment. However, the details regarding the specific molecular and cellular mechanisms/pathways underlying BI-induced deficits remain unclear [1].

There is evidence that acupuncture is effective in treating several neurological disorders such as stroke [10], depression [11], and fatigue [12]. Acupuncture exerts its therapeutic effects by stimulating acupuncture points, and adenosine A1 receptors and nerve fibers transfer the signal [13,14]. The complicated effects of acupuncture are determined by the complexity of the human body and the disease being treated. One study reported neuroimmune system modulation following acupuncture [15]. Electroacupuncture (EA) performed immediately after middle cerebral artery occlusion prevented extensive BBB damage [16] and inhibited neuroinflammation [17], two important elements in irradiation-induced brain injury [18,19]. DU20 (baihui) and DU26 (shuigou) are common acupuncture points targeted to treat neurological diseases in clinical practice and experimental studies [16], and these points are believed to be related to the brain in Chinese traditional medicine. Therefore, we selected these two acupuncture points to study the beneficial effects of EA after BI injury. Our results suggest that EA performed immediately after BI prevents irradiation-induced cognitive impairments in rats.

## Materials and Methods

### BI

This research was approved by the Animal Care and Ethics Committee of Fudan University, China, and the experiments were performed in accordance with the 1964 Declaration of Helsinki and its later amendments. A total of 112 young adult male Sprague Dawley rats aged 6–8 weeks were purchased from the Animal Center of Fudan University. The rats were housed socially in a temperature-controlled room with free access to food and water. Artificial lighting was maintained on a 12:12-h light:dark cycle. All animals were anesthetized with an intraperitoneal (i.p.) injection of a ketamine (75 mg/kg)/xylazine (15 mg/kg) mixture and irradiated with a 6 MV X-ray linear accelerator (Primus Linear Accelerator; Siemens, Germany). The eyes, neck, and body were protected with lead shielding, so only the head was exposed to X-rays. A single dose of 22 Gy was administered at 2.5 Gy/min with a source-to-skin distance of 100 cm, and the irradiation dose was selected according to previous studies [20,21].

### EA

**Fig 1:**
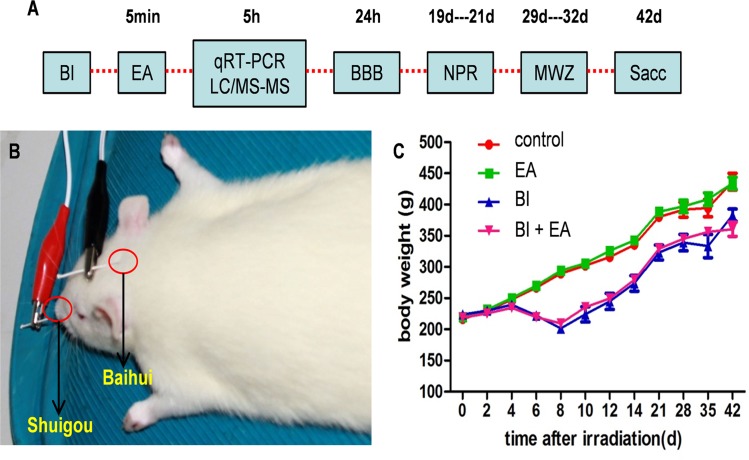
Schematic timeline of experimental procedures and body weight. (A) Young adult (6–8 weeks) Sprague–Dawley rats received BI brain irradiation (BI), and electroacupuncture (EA) was applied to rats in the EA and BI + EA groups 5 min later. Levels of amino acid neurotransmitters, their receptors, neuropeptides, cytokines, the BBB, and cognition were assessed at different time points. (B) EA was applied at the DU20 (baihui) and DU26 (shuigou) acupuncture points. (C) Body weights after BI. BI, brain irradiation; EA, electroacupuncture; LC-MS/MS, liquid chromatography and tandem mass spectrometry; BBB, blood-brain barrier; NPR, novel place recognition; MWM, Morris water maze; qRTPCR, quantitative real-time reverse transcription polymerase chain reaction; Sac, sacrifice.

Immediately after irradiation (approximately 5 min later), EA was applied for 30 min at the acupuncture points DU20 (baihui) and DU26 (shuigou) using an electrical stimulation device (HANS LH-202, Huawei Co, Beijing, China) (Fig. 1B). The wave type was dense sparse; frequency, 2/15 Hz; and intensity, 3 mA [16].

### Evans blue (EB) staining

EB (12% in saline, 4 mL/kg, Sigma, USA) was injected through the tail vein. Two hours later, rats were anesthetized with an intraperitoneal (i.p.) injection of a ketamine (75 mg/kg)/xylazine (15 mg/kg) mixture and then perfused with saline until a colorless perfusion fluid was obtained from the right atrium. The hippocampus and cortex (above the hippocampus, similar volume) were carefully dissected on ice and stored at -80°C until use. Samples were weighed, placed in a dimethylformamide solution (1 mL/100 mg), and minced into small pieces. After incubation at 60°C for 24 h, fluorescence was detected using a microplate reader (excitation at 620 nm, emission at 680 nm; Synergy4, BioTek, USA).

### Dynamic contrast-enhanced magnetic resonance imaging (DCE-MRI)

The head of an anesthetized rat was placed at the center of the animal coil of a 3.0-Tesla Tim-Trio MRI system (Siemens, Germany). A T1-weighted gradient-echo sequence (repetition time [TR]/echo time [TE] = 200 ms/12 ms) was applied to obtain nine axial images and to facilitate slice positioning. Subsequently, T2-weighted coronal images were acquired using a fast spin-echo sequence (TR/TE = 2200 ms/102.5 ms, slice thickness = 2 mm, slice gap = 2 mm). The dynamic susceptibility contrast MRI method was applied for DCE-MRI using a spin-echo echo planar imaging sequence (TR/TE = 780 ms/14.4 ms, slice thickness = 2 mm, slice gap = 2 mm). During the 10-min dynamic imaging series, contrast media (0.67 mmol/kg; Magnevist, Germany) was manually injected as a bolus by the same researcher (using an average amount of 0.2 mL in 1 s) into the tail vein, starting at the second measurement. T2 MRI and relative cerebral blood flow (CBF) maps were merged using the Nordic ICE v2.2 software (Nordic Imaging Lab AS, Norway). Six templates of regions of interest (ROIs) were contoured on the merged images, including the left and right hippocampi, cortex, and neck muscles. A dynamic enhancement curve of each ROI was obtained, and the maximum enhancement ratio (MER) was measured. To decrease the effect of the amount of contrast medium and injection velocity, the relative enhancement (RE) of the brain (regulated using the neck-muscle MER) was used to assess BBB permeability with the following equation:

$$RE=\frac{MER(brain)}{MER(muscle)}$$

### Electron microscopy

Anesthetized rats were perfused for 60 s with 0.9% saline through the left ventricle of the heart and then infused with 4% paraformaldehyde and 2% glutaraldehyde mixed in 0.1 M phosphate buffer (pH 7.4). After transcardiac perfusion, a small block (approximately 1 × 1 × 3 mm) was cut from the hippocampus. The block was further fixed in 2% phosphate-buffered saline (PBS)/glutaraldehyde, followed by 1% osmium. The tissue block was dehydrated stepwise in ethanol, block-stained with saturated uranyl acetate, embedded in Araldite, and sectioned using an Ultracut-E Ultramicrotome (Reichert-Jung, Austria). Ultrathin sections (50 nm) were examined using a JEOL transmission electron microscope (JEM-1230, Japan).

### Western blotting

The hippocampus was removed rapidly, placed on ice, and processed for western blotting. Equal amounts of protein (30 μg) were loaded onto sodium dodecyl sulfate polyacrylamide gel electrophoresis (SDS-PAGE) gels, separated by electrophoresis, and transferred onto nitrocellulose membranes. Membranes were blocked and probed overnight at 4°C with the following primary antibodies: anti-synaptophysin (1:250, mouse, Abcam, UK), and anti-glyceraldehyde 3-phosphate dehydrogenase (GAPDH; 1:500, mouse, Santa Cruz, USA). Immunoblots were processed with the appropriate secondary antibodies (1:8000, mouse, Santa Cruz) at room temperature for 1 h, and bands were visualized using the ECL Plus chemiluminescence reagent kit (Amersham Bioscience, UK). Quantification was performed via an optical density method using ImageJ software (National Institutes of Health, USA). Results are expressed as the relative density to GAPDH and normalized to the mean control group value.

### Quantitative real-time reverse transcription polymerase chain reaction (qRTPCR)

Total RNA was extracted from the hippocampus using the TRIzol reagent (Invitrogen, USA). To analyze the expression levels of individual target genes, 2 mg total RNA from each rat was reverse transcribed into cDNA using the reverse transcription system (Promega, USA). qRTPCR analysis was performed using a qRTPCR detection system (iCyclerQ real-time PCR Detection System, Bio-Rad, USA) with SYBR Green (Bio-Rad). The cycling conditions were as follows: incubation for 3 min at 95°C, followed by 40 cycles of three-step PCR consisting of a 95°C step for 30 s followed by an appropriate temperature for annealing and a 72°C-extension step for 30 s. Amplifications were carried out in triplicate, and relative target gene expression was determined using the ΔΔCt method with GAPDH gene expression as an internal control.

### Neurotransmitters

Five hours after irradiation, the hippocampi of unanesthetized rats were homogenized in an equal volume mixture of methanol/H_2_O (100 mg/1 mL, 4°C). Thirty minutes later, a supernatant was obtained by centrifugation (10,000 rpm, 4°C, 10 min) and stored at -20°C until further use. Amino acid neurotransmitter (glutamate, aspartate, gamma-aminobutyric acid [GABA], taurine, and glycine) levels were measured using liquid chromatography-tandem mass spectrometry (LC-MS/MS). All standards and conventional reagents were purchased from Sigma.

### Novel place recognition (NPR) test

The NPR test is a rodent hippocampal-dependent memory test that begins with 3 days of habituation, and was performed three weeks after irradiation. Each day, rats were placed individually in the open-field arenas (70 × 70 × 40 cm high) with two toy objects that were not used in the subsequent experimental trials and were allowed to explore freely for 10 min before returning to their home cages. The familiarization phase and 5-min test phase were administered on the fourth day. For the familiarization phase, two identical plastic blocks (8 × 5 × 13 cm high) were placed at specific positions within the arena, and rats were allowed to explore the arena freely for 5 min. Then, rats were placed in a small holding cage for a 5-min interval, and one block was moved to a novel spatial position. Rats were returned to the arena for the 5-min test and allowed to freely explore the arena for 3 min. For all phases, the arena and objects were cleaned with 70% ethanol between trials to minimize odor cues. A video camera was centered above the arena, and live tracking of the animals was performed with Noldus Ethovision XT (version 7.0; Noldus Information Technology, USA). A rat was considered to be exploring a block when its head was oriented toward the block and its nose was within a 4-cm radius. The exploration ratio (time spent exploring the novel position/total time spent exploring) of the first 60 s of the 5-min test was calculated to assess spatial recognition.

### Morris water maze (MWM) testing

The MWM is a classic method of investigating spatial learning and memory in rats, and was performed five weeks after irradiation. Lesions in distinct brain regions like the hippocampus, striatum, basal forebrain, cerebellum, and cerebral cortex impair MWM performance. This test consisted of three phases: acquisition, probe testing, and reversal learning testing. During the acquisition phase (2 days, four trials/day), the platform was hidden under water (1.5 cm) in a 200-cm diameter pool. The maximum trial duration was 60 s, after which rats were manually guided to the platform where they were to remain for 30 s. On day 3 (probe test), the platform was removed, and rats were allowed to swim freely for 60 s. After the probe trial, the platform was returned to the same place, and three regular acquisition trials were performed. For the reversal learning test (day 4), the platform was moved to a new quadrant, and two 60-s trials were performed. All performances were monitored with the ANY-Maze automated video-tracking system (Stoelting, USA).

### Immunohistochemistry and immunofluorescence

The perfusion of anesthetized rats was performed through the left ventricle with 0.9% saline, followed by 4% paraformaldehyde. Rat brains were retrieved, postfixed for 3 days, and cryoprotected in a 30% sucrose solution. Coronal sections containing the hippocampus were cut at a thickness of 40 μm. Every 12th section was collected in one well of a 24-well plate and stored at -20°C in tissue cryoprotectant solution. For the quantitative detection of doublecortin (DCX) labeling, every 12th section from each rat (8 sections in total) was collected and washed with PBS twice for 5 min each, then treated with 0.3% H_2_O_2_ at room temperature for 30 min. After blocking with 5% donkey serum containing 0.05% Triton X-100 for 1 h, the sections were incubated with a mouse anti-DCX antibody (1:200; Santa Cruz) at 4°C overnight. Sections were then incubated for 1 h with a secondary antibody (1:200, biotinylated rabbit anti-goat, Jackson Immunoresearch, USA), followed by signal amplification with an avidin-biotin complex and visualization by 3,3-diaminobenzidine catalysis. Whole dentate gyrus reconstructions were generated using an E600FN Neurolucida (Nikon, Japan) equipped with a 20× objective. DCX-positive (DCX+) cells in the subgranular cell (SGC) layers were counted using ImageJ software, and the total number was estimated by multiplying the number of cells counted in every 12th section by 12.

After washing in PBS, sections were incubated with anti-synaptophysin (1:200, Abcam) antibodies in 5% donkey serum in PBS at 4°C overnight. After washing in PBS, the sections were incubated with secondary antibodies (1:400, mouse, fluorescein isothiocyanate, Invitrogen). Sections were counterstained with 4',6-diamidino-2-phenylindole (DAPI, Sigma) to identify nuclei and observed under a confocal laser scanning microscope (Zeiss LSM 510, Germany).

### Statistical analysis

Results are expressed as the mean ± the standard error of the mean (SEM). Differences between mean values were determined using one-way analyses of variance (ANOVAs) with Tukey tests for posthoc comparisons. *P* < 0.05 was considered statistically significant. GraphPad Prism5 software (GraphPad Software, USA) was used to analyze data and plot graphs.

## Results

### EA protects the BBB after irradiation

**Fig 2:**
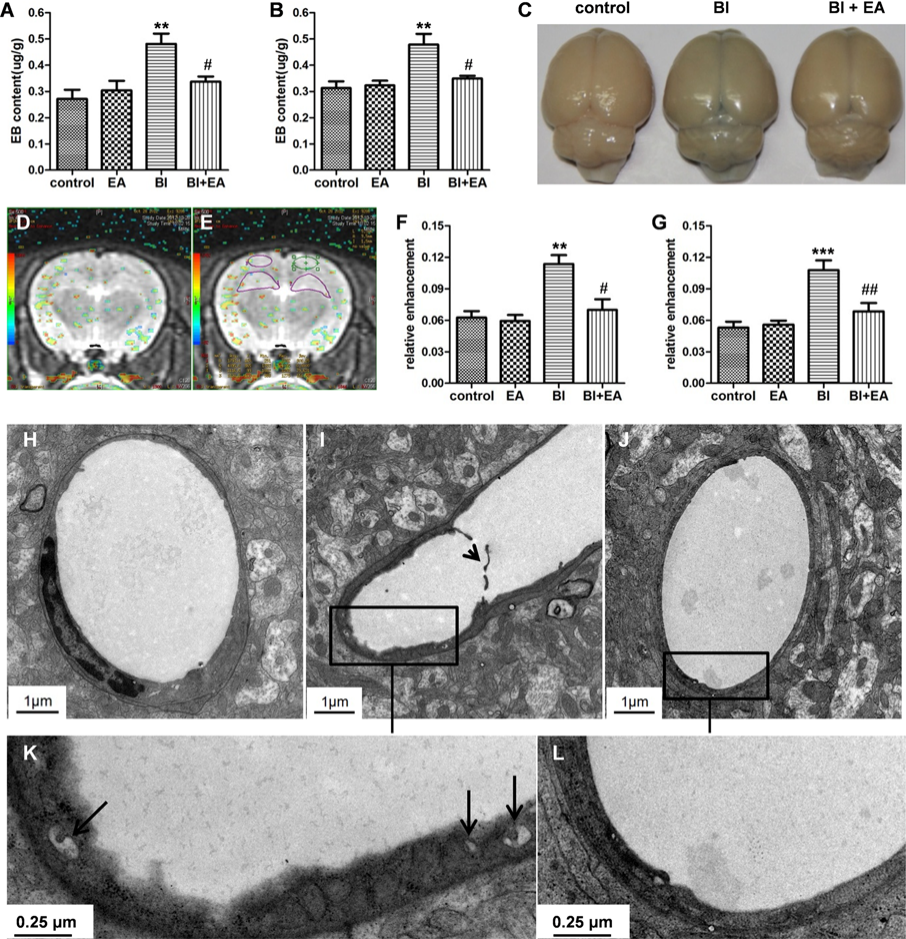
EA protects the BBB after brain irradiation. (A, B) BBB permeability in the hippocampus and cortex was detected by Evans blue staining 24 h after brain irradiation (n = 5). (C) Whole brains after Evans blue injection. (D) Merged T2 MRI and relative CBF maps. (E) The hippocampus and cortex were contoured on the merged image. (F, G) BBB integrity in the hippocampus and cortex was detected by DCE-MRI (n = 7). (H–L) Electron microscopy image of the hippocampal microvasculature (control: H, BI: I and K, BI + EA: J and L) showing membrane disruption (I, short black arrow) and vesicle increment (K, long black arrow) in the BI group. Short arrow: shedding membrane; long arrow: vesicles. Data are shown as means ± SEM. *P < 0.05, **P < 0.01, and ***P < 0.001 compared with the control group. #*P* < 0.05 and ##*P* < 0.01 compared with the BI groups.

To test whether performing EA immediately after irradiation is beneficial, we first examined BBB permeability, which can predict irradiation-induced cognitive dysfunction [18]. EB levels in the hippocampus and cortex were significantly increased 24 h after BI (BI vs. control, *P* < 0.01, Fig. 2A and 2B, respectively). EA performed immediately after irradiation decreased EB content in both the hippocampus and cortex (BI vs. BI + EA, *P* < 0.05, Fig. 2A and 2B, respectively). After 24 h of EB administration, the brain color of the BI group was bluer than that of the control group, while the brain color in the BI + EA group was less blue than that of the BI group (Fig. 2C). Similar results were obtained with DCE-MRI. The RE values of the hippocampus and cortex increased 24 h after BI (BI vs. control, *P* < 0.01, Fig. 2F; *P* < 0.001, Fig. 2G), but EA prevented these increases (BI + EA vs. BI, *P* < 0.05, Fig. 2F; *P* < 0.01, Fig. 2G).

To determine the protective mechanisms of EA on the BBB after BI, electron microscopy was used to explore ultrastructural changes in the hippocampal BBB. Microscopic images from control rats showed normal endothelial structures with a well-connected basal lamina (Fig. 2H). In irradiated animals, many vesicles were observed in the cytoplasm (Fig. 2K), and some membranes were disrupted (Fig. 2I). Additionally, the endothelial structure of the BI + EA group was similar to that of the control group (Fig. 2J, 2L).

### EA modulates cytokine mRNA expression after irradiation

**Fig 3:**
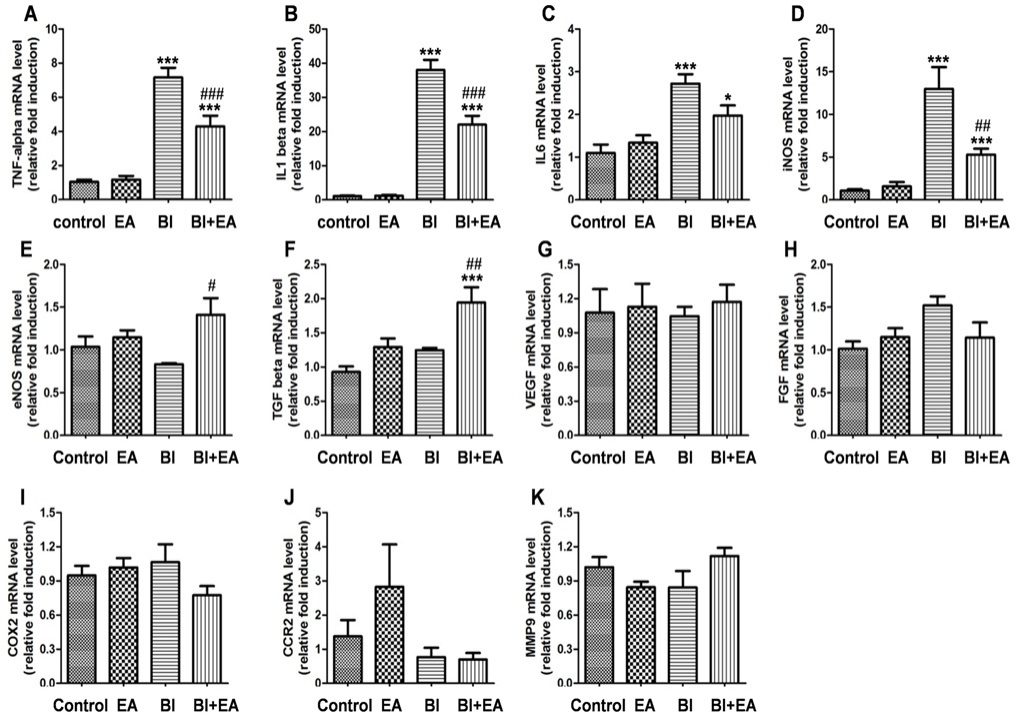
EA modulates the mRNA expression of cytokines after brain irradiation. The mRNA expressions of TNF-α (A), IL-1β (B), IL-6 (C), iNOS (D), eNOS (E), TGF-β (F), VEGF (G), FGF (H), COX2 (I), CCR2 (J), and MMP9 (K) were detected by qRTPCR 5 h after brain irradiation (n = 6). Data are shown as means ± SEM. *P < 0.05 and ***P < 0.001 compared with the control group. #*P* < 0.05, ##*P* < 0.01, and ###*P* < 0.001 compared with the BI group.

Tumor necrosis factor-alpha (TNF-α) is a key factor in BBB destruction in a brain-irradiation injury mouse model [22]; thus, we assessed the expression of TNF-α and other cytokines in the hippocampus 5 h after BI. We observed that irradiation significantly increased the mRNA levels of the cytokines TNF-α (BI vs. control, *P* < 0.001, Fig. 3A), interleukin-1 beta (IL-1β; BI vs. control, *P* < 0.001, Fig. 3B), IL-6 (BI vs. control, *P* < 0.001, Fig. 3C), and inducible nitric oxide synthase (iNOS; BI vs. control, *P* < 0.001, Fig. 3D). However, irradiation did not affect the gene expression of the cytokines endothelial NOS (eNOS; Fig. 3E), transforming growth factor beta (TGF-β, Fig. 3F), vascular endothelial growth factor (VEGF, Fig. 3G), fibroblast growth factor (FGF, Fig. 3H), cyclooxygenase-2 (COX2, Fig. 3I), C-C chemokine receptor type 2 (CCR2, Fig. 3J), and matrix metallopeptidase 9 (MMP9, Fig. 3K). mRNA expression levels of TNF-α (BI + EA vs. BI, P < 0.001, Fig. 3A), IL-1β (BI + EA vs. BI, *P* < 0.001, Fig. 3B), and iNOS (BI + EA vs. BI, *P* < 0.01, Fig. 3E) decreased when EA was applied immediately after irradiation, but remained higher than their respective control levels (TNF-α: BI + EA vs. control, *P* < 0.001, Fig. 3A; IL-1β: BI + EA vs. control, *P* < 0.001, Fig. 3B; iNOS: BI + EA VS control, *P* < 0.001, Fig. 3D). Interestingly, EA enhanced the mRNA expression levels of eNOS (BI + EA vs. BI, *P* < 0.05, Fig. 3E) and TGF-β (BI + EA vs. BI, *P* < 0.01; BI + EA vs. control, *P* < 0.001; Fig. 3F), which were not altered after irradiation.

### EA regulates neurotransmitter receptors and neuropeptides after irradiation

**Fig 4:**
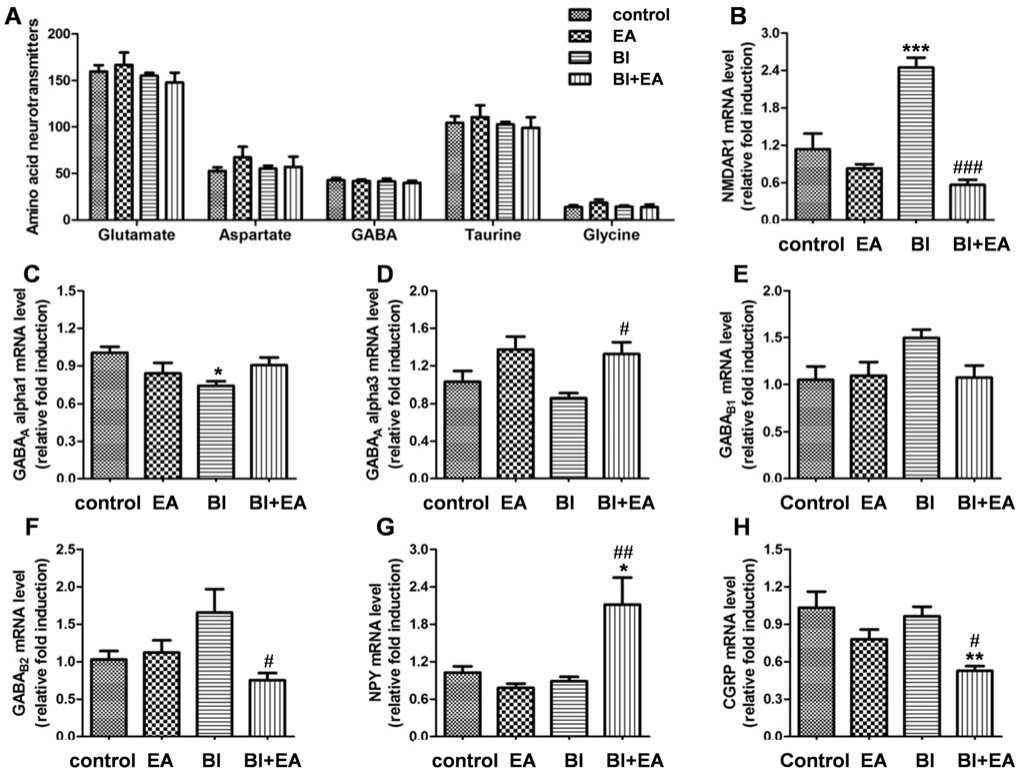
EA regulates neuronal function after brain irradiation. (A) The total content of excitatory and inhibitory amino acid neurotransmitters in the hippocampus was detected by LC-MS/MS 5 h after brain irradiation. (B–G) The mRNA expressions of NMDAR1 (B), GABA receptors (GABAAα1, C; GABAAα3, D; GABAB1, E; and GABAB2, F) and neuropeptides (NPY, G and CGRP, H) were detected by qRTPCR 5 h after brain irradiation (n = 6). Data are shown as means ± SEM. *P < 0.05 and **P < 0.01 compared with the control group. #*P* < 0.05 and ##*P* < 0.01 compared with the BI group.

To test whether EA affects neuronal function via effects on neurotransmission, we first determined the total contents of excitatory/inhibitory amino acid neurotransmitters in the hippocampus by LC-MS/MS. No changes were found in the amino acid neurotransmitter levels 5 h after irradiation (glutamate, *P* = 0.5565; aspartate, *P* = 0.6261; GABA, *P* = 0.8441; taurine, *P* = 0.8436; glycine, *P* = 0.4987; Fig. 4A). We subsequently detected the expression of subsets of receptors for glutamate and GABA. EA inhibited the increase in NMDAR1 expression 5 h after irradiation (BI vs. control, *P* < 0.001; BI + EA vs. BI, *P* < 0.001; Fig. 4B), restored the decrease in GABA_A_ α1 expression after irradiation (BI vs. control, *P* < 0.05; Fig. 4C), increased GABA_A_ α3 expression after irradiation (BI + EA vs. BI, *P* < 0.05; Fig. 4D), and decreased GABA_B2_ expression (BI + EA vs. BI, *P* < 0.05; Fig. 4F). The expression of two neuropeptides was also examined. EA increased neuropeptide Y expression (NPY; BI + EA vs. control, *P* < 0.05; BI + EA vs. BI, *P* < 0.01; Fig. 4G) and decreased postirradiation calcitonin gene-related peptide expression (CGRP; BI + EA vs. control, *P* < 0.01; BI + EA vs. BI, *P* < 0.05; Fig. 4H).

### EA ameliorates radiation-induced cognitive dysfunction

**Fig 5:**
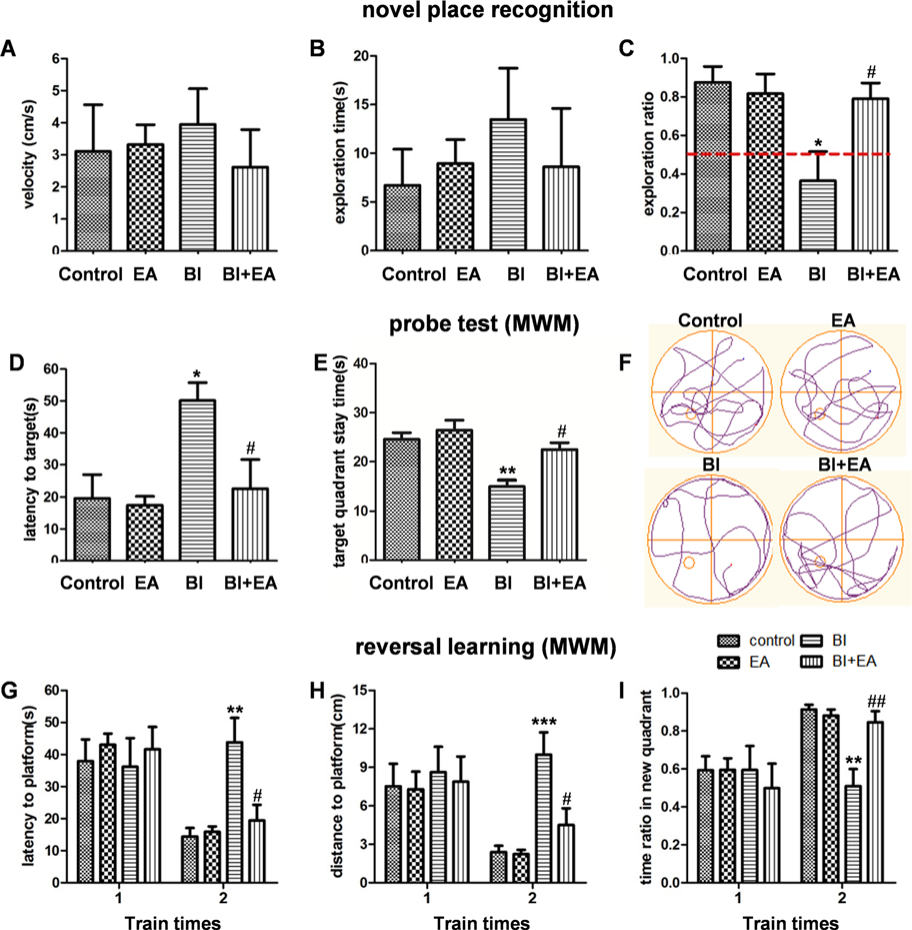
EA ameliorates radiation-induced cognitive dysfunction. Velocity (A) and exploring time (B) during the familiarization phase of the novel place recognition test. (C) Exploring ratio during the first 60-s for a novel place in the 5-min test. (D) Latency to find the target (the platform’s previous position), (E) the time in the target quadrant, and (F) the track maps during the probe test of the Morris water maze. (G) Latency to find the platform, (H) distance to the platform, and (I) ratio of time spent in the new quadrant in the reversal learning test of the Morris water maze (n = 8). Data are shown as means ± SEM. *P < 0.05, **P < 0.01, and ***P < 0.001 compared with the control group. #*P* < 0.05 and ##*P* < 0.01 compared with the BI group.

As EA reversed some of the early changes that occur after BI injury, it may also ameliorate irradiation-induced cognitive impairment. To test this hypothesis, NPR and MWM tests were performed to assess hippocampus-dependent cognitive function. During the familiarization phase of the NPR test, analyses of the velocity and total time spent exploring the objects revealed no significant differences between groups (*P* = 0.7539, Fig. 5A; *P* = 0.6326, Fig. 5B). During the 5-min test phase, animals showed significant discrimination between exploring novel and familiar places (*P* = 0.0304, Fig. 5C). The exploring ratio of the BI group was much lower than that of the control group (*P* < 0.05, Fig. 5C); however, the BI + EA group exhibited more interest in exploring novel places compared with the irradiation group (*P* < 0.05, Fig. 5C), suggesting that EA protected cognitive function as assessed with the NPR.

During the MWM acquisition phase, there were no significant group differences in the latency to find the hidden platform. In the probe test, rats in the BI group spent more time locating the target compared to rats in the control group (control vs. BI, *P* < 0.05, Fig. 5D), and the time in the target quadrant also decreased (control vs. BI, *P* < 0.01, Fig. 5E). However, these two indexes were not significantly different between the BI + EA and control groups. Nevertheless, compared to rats in the BI group, those in the BI + EA group spent less time finding the target location (*P* < 0.05, Fig. 5D) and spent more time in the target quadrant (*P* < 0.05, Fig. 5E). All animals performed similarly during the first trial of the reversal learning test; however, during the second trial, rats in the BI group swam for longer and covered a greater distance (BI vs. control, *P* < 0.01, Fig. 5G; *P* < 0.001, Fig. 5H) before they found the hidden platform (placed in a different quadrant than the acquisition phase) and spent less time in the new quadrant (BI vs. control, *P* < 0.01, Fig. 5I). Moreover, the BI + EA group performed better than the BI group (*P* < 0.05, Fig. 5G; *P* < 0.05, Fig. 5H; *P* < 0.01, Fig. 5I).

### EA does not restore hippocampal neurogenesis postirradiation

**Fig 6:**
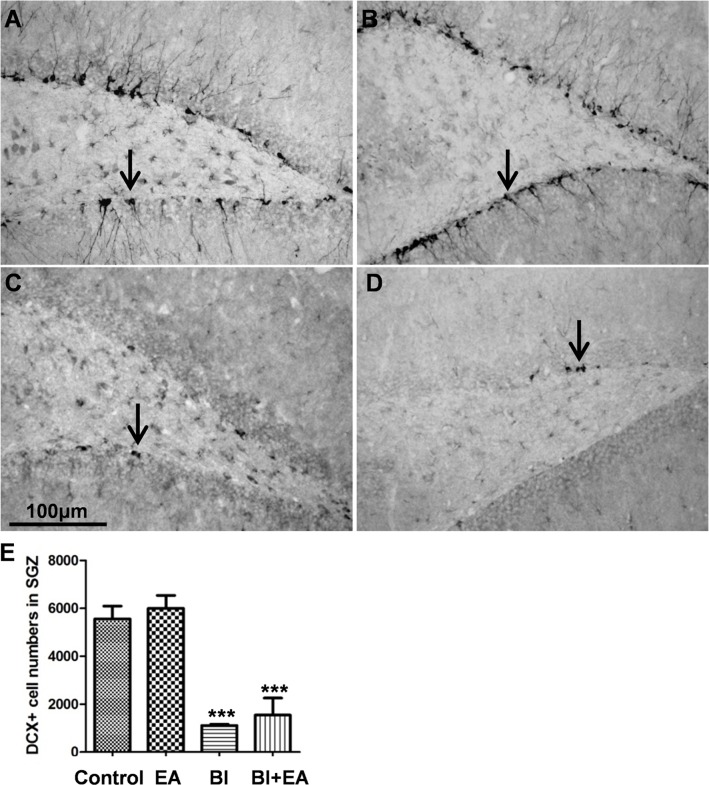
EA does not restore hippocampal neurogenesis in the dentate gyrus after brain irradiation. DCX+ cells (black arrow) in the subdentate gyrus zone were decreased in both the 22 Gy and 22 Gy + EA group at 6 weeks postirradiation (A: control group; B: EA group; C: 22 Gy group; D: 22 Gy + EA group). Data are shown as means ± SEM, n = 4. ***P < 0.001 compared with the control group.

To determine whether the EA-induced protection of cognition after irradiation was dependent on hippocampal neurogenesis, we quantitatively detected DCX+ immature neurons in the subdentate gyrus. Six weeks after BI, the numbers of DCX+ cells were significantly decreased in both the BI and BI + EA groups (BI vs. control, *P* < 0.001; BI + EA vs. control, *P* < 0.001; Fig. 6E); however, the values in these two groups were not significantly different.

### EA prevents the decrease in synaptophysin expression in the hippocampus following irradiation

**Fig 7:**
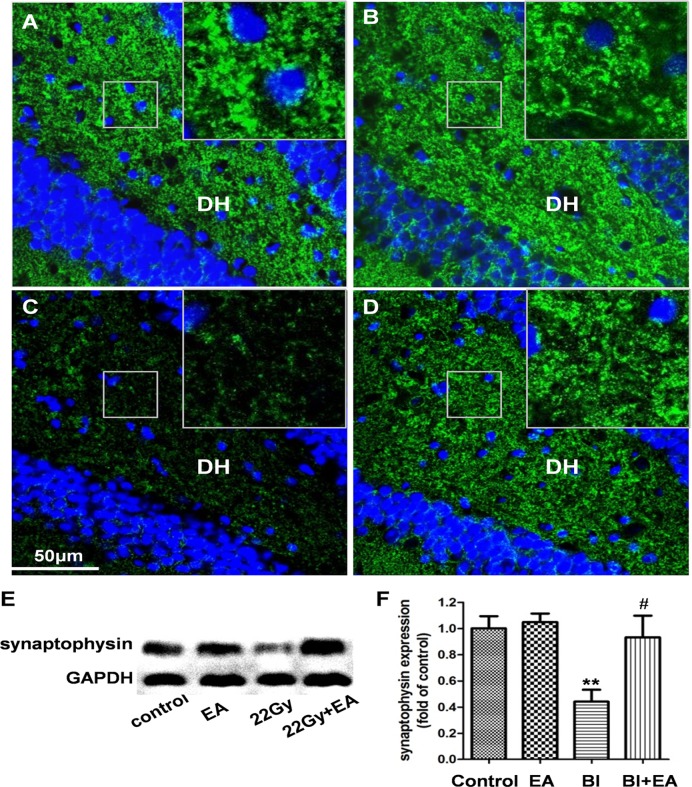
EA prevents decreased hippocampal synaptophysin expression after brain irradiation. Fewer synaptophysin punctae (green) in the dentate hilus (DH) were only observed in the BI group (A: control group; B: EA group; C: 22 Gy group; D: 22 Gy + EA group). (E, F) Synaptophysin expression in the hippocampus was protected by EA after brain irradiation. Data are shown as means ± SEM, n = 4. **P < 0.01 compared with the control group, #*P* < 0.05 compared with the BI group.

To explore whether the EA-induced protection of cognition after irradiation was dependent on synaptic density, we assessed expression of the presynaptic marker synaptophysin in the hippocampus [23]. Six weeks after irradiation, synaptophysin expression was decreased by 56% in the BI group (BI vs. control, *P* < 0.01, Fig. 7E and 7F); however, the BI + EA group expressed higher synaptophysin levels than the BI group (*P* < 0.05, Fig. 7E and 7F). Immunofluorescence images also showed fewer synaptophysin punctae in the dentate hilus (DH) of the BI group compared to the control group (Fig. 7A and 7C), whereas the BI + EA group synaptophysin punctae intensity was similar to that observed in the control group (Fig. 7D).

## Discussion

The BBB selectively controls central nervous system (CNS) homeostasis by affecting specific structural and biochemical features of endothelial cells, pericytes, and astrocyte endfeet. BBB damage by BI, which is caused by endothelial apoptosis [24], vesicle increment [25], and tight junction loss [26], may predict irradiation-induced cognitive dysfunction [18]. Our experiments showed that BBB integrity in both the hippocampus and cortex was protected by performing EA immediately after irradiation (Fig. 2A–C, 2F, and 2G). Additionally, EA inhibited irradiation-induced membrane shedding (Fig. 2I) and vesicle increment (Fig. 2K). EA performed immediately after irradiation decreased the mRNA expression of TNF-α (Fig. 3A), IL-1β (Fig. 3B), and iNOS (Fig. 3D), indicating that EA inhibited neuroinflammation. Interestingly, when combined with irradiation, EA increased mRNA expression levels of eNOS (Fig. 3E) and TGF-β (Fig. 3F), both of which play a central role in angiogenesis [27,28]. Together, our results suggest that EA protects the BBB after BI by inhibiting neuroinflammation and upregulating angiogenic cytokine expression.

Our data showed that EA decreased NMDAR1 (Fig. 4B) and GABA_B2_ expression (Fig. 4F) and increased GABA_A_ α3 expression (Fig. 4D), although the total contents of excitatory and inhibitory amino acid neurotransmitters in the hippocampus did not change at 5 h postirradiation (Fig. 4A). NMDAR1 downregulation may mitigate glutamate excitotoxicity after BI [29]. GABA_A_ receptors inhibit the microglial inflammatory response via a Cl^−^ influx [30], and GABA_B_ receptors inhibit GABA release by preventing Ca^2+^ influx at the presynaptic membrane [31]. Our findings suggest that EA may attenuate neuroinflammation by decreasing the inhibition of GABA release and increasing GABA_A_ receptor expression. Moreover, the upregulation of NPY (Fig. 4G), which attenuates glutamate release [32] and inhibits IL-1 β-induced phagocytosis by microglia [33], may contribute to EA’s anti-inflammatory action. These findings will pave the way for further research into the multiple pathways involved in EA protection.

Administration of peroxisomal proliferator-activated receptor (PPAR) agonist pioglitazone before, during, and for 4 weeks postirradiation can prevent cognitive impairments, but starting administration 24 h after BI is ineffective [34]. The administration of pioglitazone [34] and the AT1 receptor antagonist (L-158,809) [35] for a short time (4 or 5 weeks) postirradiation is sufficient to prevent cognitive impairments. Collectively, the existing evidence suggests that treatment must occur during the early phase to prevent irradiation-induced cognitive impairments. Neural excitotoxicity, neuroinflammation, cell apoptosis, and BBB disruption are significant features during the early postirradiation phase. Ameliorating neural excitotoxicity with memantine [36] and inhibiting neuroinflammation with a PPAR agonist [37] have been shown to successfully prevent cognitive dysfunction, and BBB disruption can predict cognitive impairments [8]. However, neither abrogation of cell apoptosis [38] nor intracellular adhesion molecule (ICAM)-1 knockout [39] alter later injury. Therefore, some but not all early changes have causative roles in cognitive dysfunction, and the molecular cascades between the early and late changes are complicated. Here, we showed that EA performed immediately after irradiation can prevent irradiation-induced cognitive impairments. Because the effects of both irradiation and EA are complicated, the exact molecular mechanisms were previously unknown. The present findings indicate that changes, such as BBB protection and decreased expression of neuroinflammatory cytokines and NMDA receptors may prevent the development of cognitive dysfunction.

Similar to our study (Fig. 6B), another group reported that the PPAR delta agonist GW0742 prevented the irradiation-induced acute hippocampal inflammatory response, but did not attenuate decreased hippocampal neurogenesis [40]. This suggests that it is not the number of neural stem cells that is critical [38]; rather, their functionality plays a crucial role in decreasing hippocampal neurogenesis after BI, which is caused by incompletely repaired DNA damage [20], microvascular angiogenesis disruption, neuroinflammation, and oxidative stress [41]. Because pretreatment with EA attenuated oxidative stress in the ischemia-reperfusion model [42] and continuous treatment with EA increased BDNF [42] and glial cell-line-derived neurotrophic factor expression [43], future studies should apply an optimized EA protocol after lower dose irradiation to test its ability to restore hippocampal neurogenesis.

Similar to EA, CCR2 deficiency [44], L-158,809 [45], and ramipril [46] prevent cognitive impairments, but do not influence neurogenesis. Usually, the role of hippocampal neurogenesis in learning and memory is explored by decreasing neurogenesis with irradiation, antimitotic drugs, and mutational approaches, whereas neurogenesis is increased by environmental enrichment and voluntary running. It should be noted that these approaches do not only affect neurogenesis; for example, irradiation also affects neuronal architecture [6]. Thus, negative reports regarding hippocampal neurogenesis should be considered [47,48]. For example, the toxin methyl azoxymethane acetate decreases neurogenesis; however, this antimitotic agent only impairs one kind of hippocampal-dependent memory (trace fear conditioning), while two forms of hippocampal-dependent learning and memory (contextual fear conditioning and spatial navigation learning in the MWM) are not affected [48]. Interestingly, X-ray irradiation and genetic overexpression of follistatin, both of which severely impair hippocampal neurogenesis, prolong the hippocampus-dependent periods of remote contextual fear memory, whereas running-wheel exercises that promote hippocampal neurogenesis speed up the decay rate of this kind of memory [47]. Therefore, the exact role of hippocampal neurogenesis in cognition warrants further research.

Long-lasting reductions in dendritic complexity and spine density, together with alterations in spine morphology and synaptic protein composition, are observed even after a very low irradiation dose (1 Gy) [6]. In the present study, synaptophysin expression was used to assess postirradiation neuronal connectivity impairments. Synaptophysin, a 38-kDa glycoprotein localized in synaptic vesicle membranes, is important for docking, fusion, and endocytosis and is a useful presynaptic marker [23]. EA restored synaptophysin expression, indicating that EA prevented synaptic loss after irradiation. Given the important role of structural plasticity in learning and memory [49], it is possible that synaptic loss, instead of hippocampal neurogenesis, contributes to BI-induced cognitive impairments [50]. However, further studies are needed to test this hypothesis.

EA is an economic and easy-to-administer technique with few adverse effects and has been efficacious for some cancer therapy-related side effects including fatigue [12] and chronic xerostomia [51]. Our results demonstrate that EA can also prevent irradiation-induced cognitive impairments. A clinical trial should be designed to test the efficacy of this ancient treatment in patients undergoing BI. It should be noted that EA protected the BBB after BI, which may influence chemotherapy delivery to the CNS. However, the BBB studied here was in normal brain tissue as opposed to brain tumor tissue. Thus, whether EA will affect local tumor control must be assessed in future studies.

## Conclusions

Taken together, our results suggest that EA applied immediately after BI can protect the BBB, inhibit the expression of neuroinflammatory cytokines, upregulate the levels of angiogenic cytokines, and modulate neuronal function during the acute phase, while preventing the onset of cognitive impairments in the delayed phase. EA-mediated protection of cognition was not dependent on hippocampal neurogenesis; rather, it reduced the loss of synaptophysin expression. Future studies are required to determine the protective effects of EA on hippocampal neurogenesis after EA parameter optimization and/or irradiation dose reduction. Clinical trials should also be performed to test the efficacy of this treatment.
